# Impact of contouring methods on pre-treatment and post-treatment dosimetry for the prediction of tumor control and survival in HCC patients treated with selective internal radiation therapy

**DOI:** 10.1186/s13550-021-00766-x

**Published:** 2021-03-09

**Authors:** Guillaume Nodari, Romain Popoff, Jean Marc Riedinger, Olivier Lopez, Julie Pellegrinelli, Inna Dygai-Cochet, Claire Tabouret-Viaud, Benoit Presles, Olivier Chevallier, Sophie Gehin, Matthieu Gallet, Marianne Latournerie, Sylvain Manfredi, Romaric Loffroy, Jean Marc Vrigneaud, Alexandre Cochet

**Affiliations:** 1 Department of Nuclear Medicine, Centre Georges-François Leclerc, Dijon, France; 2 ImViA Laboratory, IFTIM Team, EA 7535, University of Burgundy, Dijon, France; 3 Department of Vascular and Interventional Radiology, University Hospital Dijon, Dijon, France; 4 Department of Gastroenterology, University Hospital Dijon, Dijon, France

**Keywords:** Radioembolization, Hepatocellular carcinoma, Dosimetry

## Abstract

**Introduction:**

The aim of this study was to evaluate the impact of the contouring methods on dose metrics and their predictive value on tumor control and survival, in both situations of pre-treatment and post-treatment dosimetry, for patients with advanced HCC treated with SIRT.

**Methods:**

Forty-eight patients who underwent SIRT between 2012 and 2020 were retrospectively included in this study. Target volumes were delineated using two methods: MRI-based contours manually drawn by a radiologist and then registered on SPECT/CT and PET/CT via deformable registration (Pre-C_MRI_ and Post-C_MRI_), ^99m^Tc-MAA-SPECT and ^90^Y-microspheres-PET 10% threshold contouring (Pre-C_SPECT_ and Post-C_PET_). The mean absorbed dose (Dm) and the minimal absorbed dose delivered to 70% of the tumor volume (D70) were evaluated with both contouring methods; the tumor-to-normal liver uptake ratio (TNR) was evaluated with MRI-based contours only. Tumor response was assessed using the mRECIST criteria on the follow-up MRIs.

**Results:**

No significant differences were found for Dm and TNR between pre- and post-treatment. TNR evaluated with radiologic contours (Pre-C_MRI_ and Post-C_MRI_) were predictive of tumor control at 6 months on pre- and post-treatment dosimetry (OR 5.9 and 7.1, respectively; *p* = 0.02 and 0.01). All dose metrics determined with both methods were predictive of overall survival (OS) on pre-treatment dosimetry, but only Dm with MRI-based contours was predictive of OS on post-treatment images with a median of 23 months for patients with a supramedian Dm versus 14 months for the others (*p* = 0.04).

**Conclusion:**

In advanced HCC treated with SIRT, Dm and TNR determined with radiologic contours were predictive of tumor control and OS. This study shows that a rigorous clinical workflow (radiologic contours + registration on scintigraphic images) is feasible and should be prospectively considered for improving therapeutic strategy.

## Introduction

Hepatocellular carcinoma (HCC) is the most common form of primary liver cancer, the sixth for cancer incidence and the fourth for cancer death worldwide [1]. Moreover, many patients are diagnosed at an advanced stage. Many treatments have been developed depending on the stage of the disease, ranging from surgery to radiofrequency ablation, transarterial chemoembolization (TACE), selective internal radiation therapy (SIRT) and systemic treatments [2].

SIRT with ^90^Y microspheres is an effective and safe option for the treatment of advanced hepatocellular carcinoma (HCC), and its use is developing rapidly [3, 4]. This treatment is based on the fact that tumor vascularization is mainly arterial as opposed to hepatic vascularization. Several studies have reported the effectiveness of SIRT, with a good tumor response and safety profile. However, no superiority in terms of survival was found when compared with TACE or sorafenib [5–8].

As SIRT is a radiotherapy treatment approach, it is clear that dosimetry must be taken into account, as it has been shown that absorbed doses have a good correlation with tumor response or survival [9–13]. Moreover, personalized predictive dosimetry is now an essential prerequisite in SIRT to optimize absorbed dose delivery and find the optimal balance between efficacy and treatment-related complications [14]. Predictive dose metrics can be obtained on pre-therapeutic liver perfusion scanning performed after selective arterial injection of ^99m^Tc-MAA (macro-aggregated albumin) [10, 15–17]. Absorbed dose actually delivered (post-treatment dosimetry) can be obtained with post-therapeutic ^90^Y-microspheres PET/CT imaging, provided that ^90^Y quantitative capabilities has been validated for the system used [18].

In both situations, as in all image-based dosimetric approaches, the choice of the contouring method may have a major influence on dose metrics [19].

There have been several approaches to delineate target regions in the literature [20–24]. Most of them have been supporting the idea of delineating targeted regions directly on SPECT/CT or PET/CT images, trying to use the fusion between both images to have a good compromise between the scintigraphic (SPECT or PET) and the morphological volumes (CT) [13, 20, 21]. However, the tumor needs theoretically to be delineated on a high-resolution and high-contrast image (contrast-enhanced MRI or CT), whereas the voxel dosimetry is performed on the low-resolution scintigraphic images. But on one hand, truly morphological volumes are only available from diagnostic modalities (with contrast-enhanced possibilities) that are generally acquired at different time points within the course of treatment. And on the other hand, thresholding on counts is highly dependent on different characteristics in the image (contrast and volumes for example) but appears to be very simple to use in a clinical context [20, 21]. Our aim was to retrospectively study two different methods of contouring regions in order to assess their impact on tumor response and survival.

## Materials and method

### Patient’s characteristics and study protocol

Forty-eight patients with unresectable HCC treated in our institution with SIRT based on ^90^Y-microspheres injection from October 2012 to February 2020 were considered for this retrospective study. Among them, 23 received ^90^Y glass microspheres (TheraSphere; Biocompatibles UK Ltd., Surrey, England) and 25 received ^90^Y resin microspheres (SIR-Spheres; Sirtex Medical Limited, Sidney, Australia), depending on reimbursements nationwide which have varied over the years.

The inclusion criteria were as follows: clinical indication of SIRT decided by our institution’s multidisciplinary tumor board for palliative consideration, a contrast-enhanced MRI within 8 weeks prior to treatment, and lesions that could be unequivocally segmented on MRI images.

All patients underwent treatment-planning angiography combined with ^99m^Tc-MAA injection (work-up procedure) in order to estimate the lung shunt fraction, the targeting of the future treatment, and the activity of ^90^Y-microspheres to inject. This activity was either determined with the body surface area (BSA) model, the partition model (PM), or the vendor dosimetric model, using the ^99m^Tc-MAA SPECT/CT images as recommended [25, 26].

SIRT treatment was performed 2 weeks after the treatment planning by the same interventional radiologist as during the work-up and a ^90^Y-microspheres PET/CT was acquired the day after.

Treatment response to SIRT was evaluated according to the modified response evaluation criteria (mRECIST) [27, 28]. Overall survival (OS) was also evaluated. A follow-up contrast-enhanced MRI or CT was performed every 3 months after SIRT as recommended [29].

All procedures were in accordance with the ethical standards of the responsible committee on human experimentation (institutional and national) and with the Declaration of Helsinki. This manuscript was reviewed and approved by the internal ethic committee of our institution, with waiver of informed consent for this retrospective study.

### Planning angiography and ^99m^Tc-MAA SPECT/CT

Planning angiography and injection of ^99m^Tc-MAA (150 MBq) was performed by a trained interventional radiologist, according to the previously published guidelines [30]. ^99m^Tc-MAA was injected as much selectively as possible into the supplying arteries split according to the approximate perfused volumes supplied by each artery, in case there were more than two tumor-feeding arteries.

After the injection of ^99m^Tc-MAA, lung and liver planar scan and SPECT/CT tomography acquisitions were performed within one hour thereafter, using a hybrid scanner combining a dual-head gamma camera and a 16-slice CT scanner (Discovery NM/CT 670, GE healthcare, USA). SPECT acquisitions were performed to cover the whole liver and the lungs, using a low-energy/high-resolution (LEHR) collimator and setting 60 projections, 25 s per projection, matrix 128 × 128 with a cubic voxel size of 4.42 mm, automatic body contour and double energy window acquisition: 140 keV ± 10% for the emission window and 120 keV ± 5% for the scattering window. A low-dose CT scan was also acquired (120 kV, automatic tube current modulation with a noise index of 21 and a constrained maximum value of 150 mA) in order to perform attenuation correction.

Images were then reconstructed on a Xeleris workstation (GE healthcare) according to an OSEM 3D algorithm with 4 iterations, 10 subsets, attenuation, scatter and resolution recovery corrections, and no post-reconstruction filtering.

### Selective internal radiation therapy and ^90^Y-microspheres PET/CT

The planned activity of ^90^Y-microspheres was injected through a microcatheter according to the same method as the planning angiography, with the same catheter position, by the same radiologist.

SIRT was planned 2 weeks after planning angiography and PET/CT acquired the next day on a GEMINI TF (Philips healthcare) or a Discovery MI (GE healthcare).

For both PET scanners, two bed positions centered on the liver were acquired during 40 min (20 min per bed position).

For the Gemini TF, low-dose CT scans were acquired with a 16-slice CT scanner (Brilliance 16) using the following parameters: 120 kV, longitudinal and angular tube current modulation, a pitch of 0.688 and a slice thickness and increment of, respectively, 5 mm and 2.5 mm, and the reconstructions were performed with the TOF blob-based OSEM algorithm from Philips. As ^90^Y was not available in the isotope list during the acquisition step, the list mode file was corrected post-acquisition, using the relevant branching ratio and half-life of ^90^Y. The recommended reconstruction parameters for quantitative purposes [18] were used: 4 iterations, 8 subsets, no filter. The reconstruction voxel size was 4 × 4 × 4 mm^3^.

For the Discovery MI, low-dose CT scans were acquired with a 64-slice CT scanner (Revolution EVO) using 120 kV, automatic tube current modulation with a noise index of 30 and a constrained maximum value of 300 mA, a pitch of 1.375 and a slice thickness/increment of 2.5/1.25 mm. For the PET reconstruction, as no recommendations were available for this recent digital scanner, the following parameters were used: OSEM algorithm with 2 iterations, 17 subsets, a Gaussian filter of 5 mm and a standard *Z*-axis filter. The reconstructed voxel size was 2.73 × 2.73 × 2.79 mm^3^.

For both scanners, all image corrections (random coincidences, decay, dead time, scattering and attenuation from low-dose CT scans) were applied.

### Segmentation and registration

Retrospectively, two different contouring methods were applied to the pre- and post-treatment images.

In a first method, liver and tumor contours were manually delineated by an experienced Radiologist on MRI images (SIEMENS Healthineers Magnetom 1.5T and 3T—acquisition sequence T1 VIBE 3D post-gadolinium) acquired prior to the planning angiography. In order to assess inter-operators variability, a second operator (an experienced Nuclear Medicine Physician trained in Radiology) also delineated both liver and tumor volumes. The root-mean-square (RMS) of the coefficient of variations (standard deviation/mean value) of the volumes delineated by the two operators was calculated.

Both ^99m^Tc-MAA SPECT/CT and ^90^Y-microspheres PET/CT images were then registered with the MRI, in order to use MRI-based liver and tumor contours for pre-treatment and post-treatment dose calculations (“Pre-C_MRI_” and “Post-C_MRI_” contours, respectively).

Deformable registrations were performed by a trained Medical Physicist with the help of a multi-modality deformable registration algorithm available in MIM SurePlan (v7.0.1; MIM software Cleveland, USA) [31]. It is a general use Free-Form deformation algorithm that uses a feature similarity scoring metric. It maximizes the correspondence of high-dimensional feature descriptors computed by evaluating each image voxel in the context of its neighboring voxels. In order to assess quality of registrations, the Nuclear Medicine Physician also delineated liver contour on both the pre-treatment ^99m^Tc-MAA SPECT/CT and the post-treatment ^90^Y-microspheres PET/CT images. A quantitative evaluation was then performed by computing a DICE index between the liver MRI contour and the liver CT contour from each scintigraphic modality (SPECT/CT or PET/CT). The minimum accepted DICE index was 0.85.

In a second method, ^99m^Tc-MAA-SPECT and ^90^Y-microspheres-PET images were used to create a 10%-threshold contour (‘Pre-C_SPECT_’ and ‘Post-C_PET_,’ respectively), by selecting, all voxels exhibiting an uptake higher than 10% of the maximum uptake, within a volume of interest (VOI) surrounding the liver. This threshold-based contours was chosen to be a reasonable representation of arterial perfusion of the selected liver area in the context of SIRT [21].

### Dosimetry

The absorbed dose was calculated on the ^99m^Tc-MAA-SPECT/CT (pre-treatment dosimetry) and on the ^90^Y-microspheres-PET/CT (post-treatment dosimetry) images with the contours mentioned above. Three-dimensional voxel-based dosimetry was carried out with a research workflow in MIM SurePlan. In both pre- and post-treatment dosimetry calculations, total ^90^Y-microspheres activity in the field of view was assumed to be proportional to the scintigraphic counts in a region defined by the liver plus the lungs. This relative calibration method was applied for the ^99m^Tc-MAA-SPECT/CT, and to the ^90^Y-microspheres PET scans to avoid any bias due to the inaccuracy of the absolute quantification in PET systems [18] and also to reduce the effect of taking into account two different PET systems. Doses were calculated with the local deposition method (LDM) for pre- and post-treatment images [32, 33]. This dosimetric method is based on two assumptions. First, the implant of the microspheres is permanent, leading to a fixed relative distribution of the absorbed dose. Second, the energy released by the decay of ^90^Y-microspheres in a voxel is deposited within the same voxel. This relative patient-dependent dosimetric method is described in depth in literature [21].

**Fig. 1:**
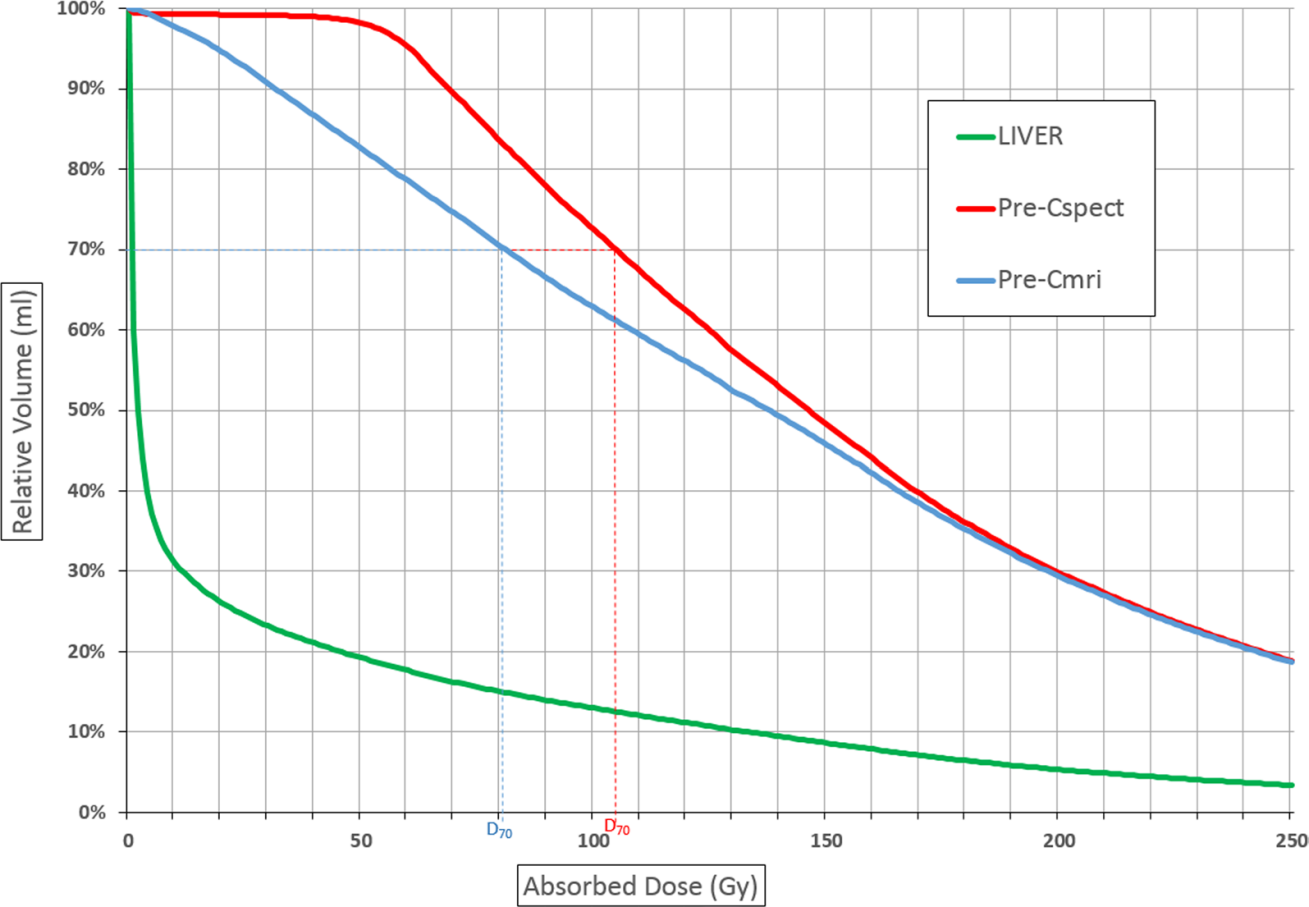
Example of dose volume histogram (DVH) computed with MiM Sureplan 7.0.1 software research workflow on pre-treatment ^99m^Tc-MAA SPECT/CT images. Abscissa is the minimal absorbed dose; ordinate is the corresponding relative volume receiving the absorbed dose. Green curve represents the liver contour; blue curve represents Pre-C_MRI_ tumor contour; red curve represents Pre-C_SPECT_ 10% threshold target contour

**Fig. 2:**
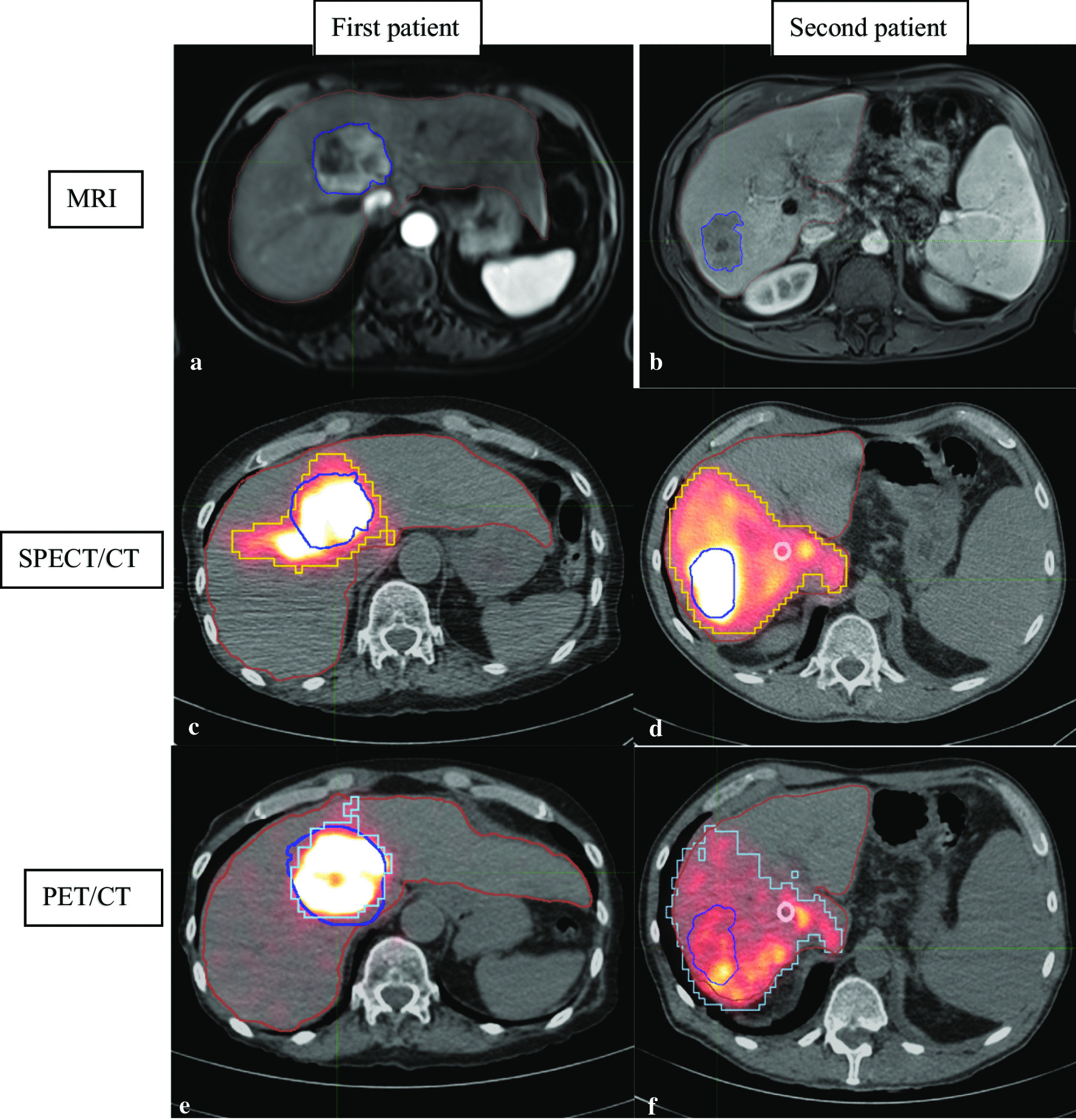
Illustration of two patients with MRI on the top (**a** and **b**) and tumors outlined in dark blue then registered on ^99m^Tc-MAA SPECT/CT and ^90^Y-microspheres PET/CT via deformable registration. Yellow and light blue contours correspond to SPECT/CT and PET/CT 10% thresholds contours, respectively. First patient (**a**, **c** and **e**) had a very high TNR determined with MRI-based contours: 10 on SPECT/CT and 19 on PET/CT. Second patient (**b**, **d** and **f**) had a very low TNR: 4 on SPECT/CT and 3 on PET/CT. In those cases, for example first patient with a very high TNR had a tumor control during 38 months and second patient with a very low TNR had a tumor control during 2 months

3D dose distribution and dose volume histogram (DVH) for each contour were computed. DVH is a 2D graph representation of the dose deposited in segmented contours (Fig. 1). From DVH, we extracted two metrics commonly used in radiotherapy to qualify dosimetric results: the mean absorbed dose (Dm) and the minimal dose covering 70% of the tumor (D70) (both expressed in Gy). They were evaluated for both contours methods. Tumor-to-normal liver uptake ratio (TNR), which is related to the hypervascularization of the tumor and the selectivity of the targeting, was also considered and was evaluated for MRI-based contours only (Fig. 2). TNR is defined as the ratio between tumor and healthy liver activity concentrations:

$${\text{TNR}} = \frac{{{\text{CNTS}}_{{{\text{tum}}}} /V_{{{\text{tum}}}} }}{{{\text{CNTS}}_{{{\text{NL}}}} /V_{{{\text{NL}}}} }},$$

where CNTS is the total number of counts in the considered contour, *V* is the contour volume in ml and tum and NL stand for the target and healthy liver contours, respectively.

### Follow-up

After treatment, most patients were assessed regularly at our institution. Follow-up contrast-enhanced MRI or CT was performed every 3 months after SIRT as recommended. For the patients unable to attend, updates were obtained from family doctors or local oncologists. Details were obtained when appropriate for both the date of tumor progression and death.

### Statistics

Continuous data were expressed as median (first quartile–third quartile) and dichotomous data as numbers (percentages).

Population was first separated in two subgroups according to the type of SIRT received (glass or resin microspheres). In each subgroup, all dose metrics obtained before treatment (based on ^99m^Tc-MAA SPECT/CT) and after treatment (based on ^90^Y-microspheres PET/CT) were compared, using Spearman’s rank correlation, Wilcoxon test and Bland–Altman analysis.

Dose metrics Dm and D70 were also compared depending on the contouring method. Median values were determined for all dose metrics (Dm, D70 and TNR) in each subgroup.

Predictive factors (tumor control at 6 months, overall survival) were assessed by gathering dose metrics from each kind of spheres according to their respective median values.

Logistic regression was performed to test for predictors of absence of progressive disease according to mRECIST [27, 28] after 6 months. All dose metrics were tested by univariate analysis, dichotomized according to the median values. For multivariate analysis, only parameters significant by univariate analysis were considered (*p* < 0.05). Kaplan–Meier and univariate Cox proportional hazard regression were also performed to determine prognostic value of dose metrics, dichotomized according to the median values (Table 1).

**Table 1:** Definition of different dose metrics evaluated

	MRI-based contours	Scintigraphic-based contours
Pre-treatment dose metrics		
Dm	Dm-Pre-C_MRI_	Dm-Pre-C_SPECT_
D70	D70-Pre-C_MRI_	D70-Pre-C_SPECT_
TNR	TNR-Pre-C_MRI_	–
Post-treatment dose metrics		
Dm	Dm-Post-C_MRI_	Dm-Post-C_PET_
D70	D70-Post-C_MRI_	D70_-_Post-C_PET_
TNR	TNR-Post-C_MRI_	–

All the tests were two-sided and a *p* value less than 0.05 was considered statistically significant.

## Results

### Patient characteristics, segmentation and registration

Patients and tumors baseline characteristics are shown in Table 2.

**Table 2:** Patients and tumors baseline characteristics. All values are median (*Q*1–Q3) or number (percentage)

	All patients (*n* = 48)	Resin microspheres (*n* = 25)	Glass microspheres (*n* = 23)	*p*
Age (y)	66 (62–73)	68 (63–74)	64 (63–72)	NS^a^
Male gender	42 (88)	23 (92)	19 (83)	NS^b^
Body surface area (m^2^)	1.9 (1.8–2.0)	1.9 (1.8–2.1)	1.9 (1.7–2)	NS^a^
Cirrhosis	37 (77)	20 (80)	16 (70)	NS^b^
Cause of cirrhosis				
Alcohol	24 (65)	14 (56)	10 (43)	NS^b^
NASH	10 (27)	7 (28)	4 (17)	NS^b^
HVC	7 (19)	4 (16)	3 (13)	NS^b^
HBV	2 (5)	2 (8)	0 (0)	NS^b^
Hemochromatosis	2 (5)	0 (0)	2 (9)	NS^b^
Child–Pugh				
A5/A6	34 (92)	18 (72)	16 (70)	NS^b^
B7	3 (8)	2 (8)	1 (4)	NS^b^
MRI-based Liver volume (ml)	1813 (1468–2358)	1912 (1505–2308)	1800 (1436–2435)	NS^a^
MRI-based Tumor volume (ml)	203 (67–545)	188 (68–312)	284 (61–738)	NS^a^
SPECT threshold-based target volume (ml)	360 (205–647)	298 (202–406)	593 (414–933)	0.002^a^
PET threshold-based target volume (ml)	386 (245–786)	365 (214–643)	398 (267–827)	NS^a^
Tumor burden (%)	11 (5–27)	8 (5–18)	16 (5–34)	NS^a^
Injected activity (GBq)	1.6 (1.1–2.6)	1.3 (1.0–1.8)	2.3 (1.4–3.3)	0.004^a^

*NASH *non-alcoholic steatohepatitis, *HBV* hepatitis B virus, *HCV* hepatitis C virus

^a^Mann and Whitney test

^b^*χ*^2^ test

No significant difference was found between resin and glass microspheres groups except for ^90^Y-microspheres injected activity (median value 1.3 vs 2.3 GBq, *p* = 0.004).

The inter-operators variability, which was computed here with the RMS of the coefficient of variations of the liver and tumor volumes delineated by the two operators, was 4.5% and 6.2%, respectively.

After registration, median Dice’s coefficient obtained between MRI-based liver contours and SPECT/CT liver contours was 0.93 [IQR 0.91–0.94] and 0.92 [IQR 0.88–0.93] between MRI-based liver contours and PET/CT liver contours.

### Comparison of pre- and post-treatment dose metrics

**Fig. 3:**
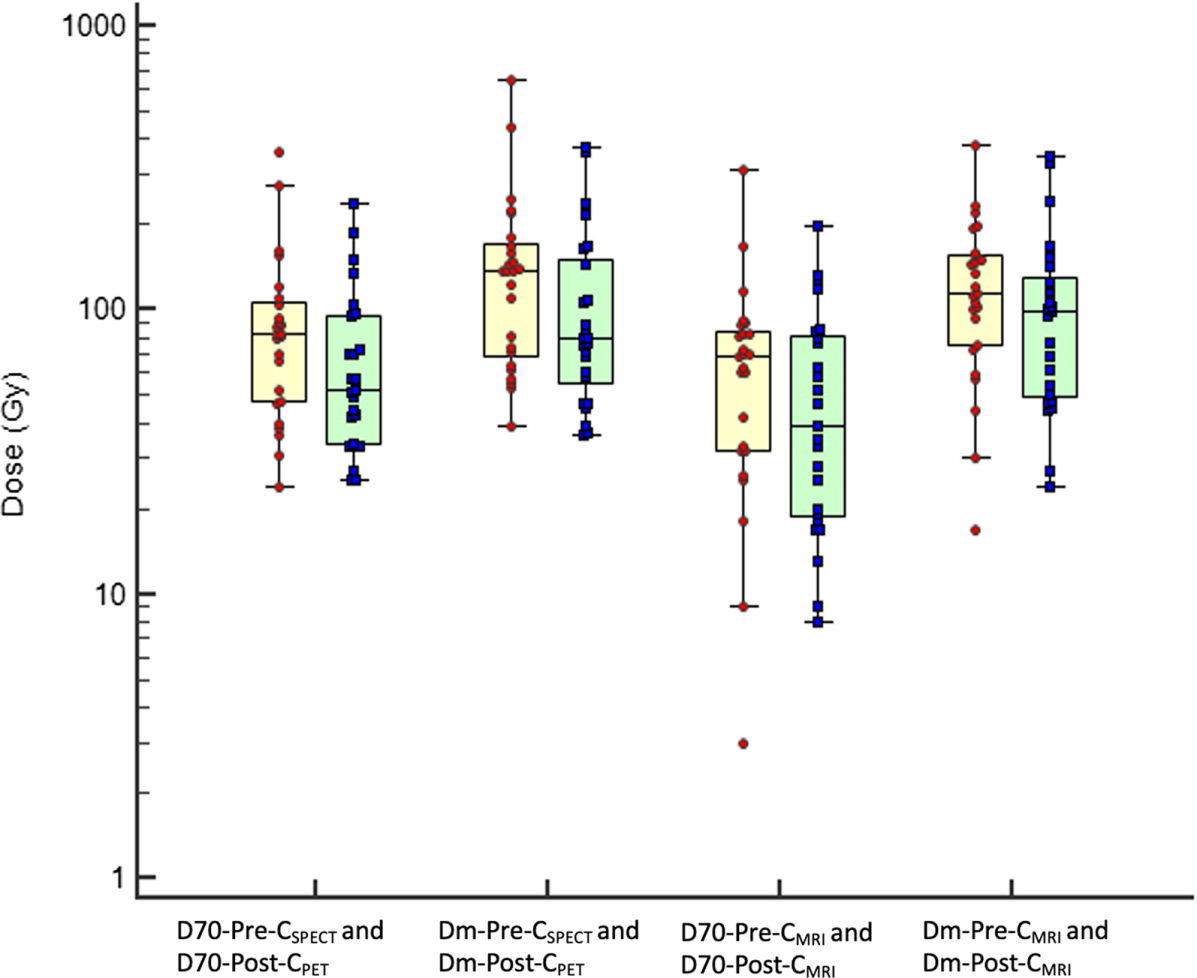
Boxplots of dose metrics based on imaging before SIRT (^99m^Tc-MAA SPECT/CT; pre-treatment dose metrics) and after SIRT (^90^Y-microspheres PET/CT; post-treatment dose metrics) in patients receiving resin microspheres

**Fig. 4:**
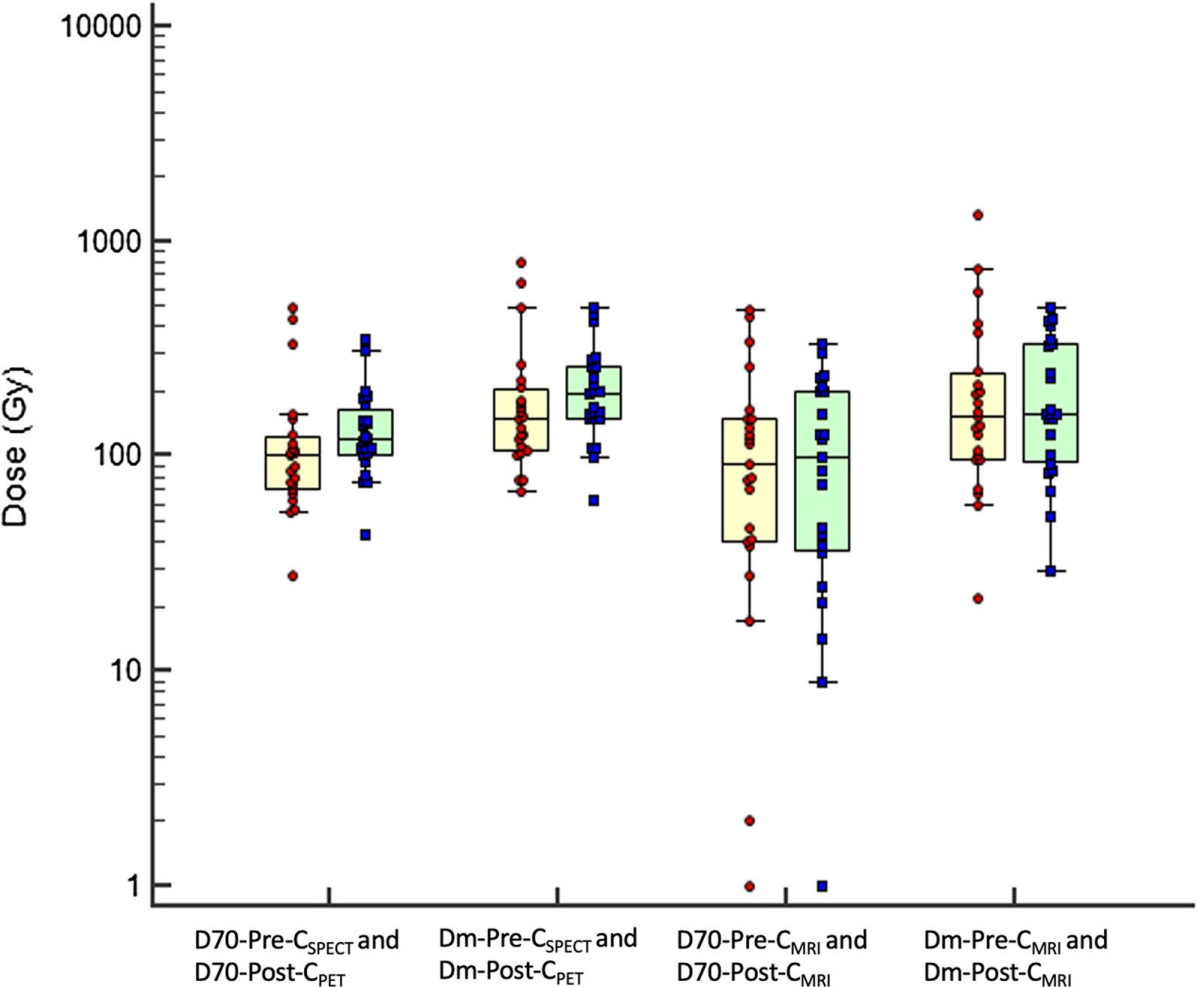
Boxplots of dose metrics based on imaging before SIRT (^99m^Tc-MAA SPECT/CT; pre-treatment dose metrics) and after SIRT (^90^Y-microspheres PET/CT; post-treatment dose metrics) in patients receiving glass microspheres

Concerning comparison of dose metrics calculated on the pre-treatment images versus post-treatment images (Table 3, Figs. 3, 4), there were no significant differences for Dm and TNR. Concerning the D70, there were also no significant differences except for D70 calculated with MRI-based contours, in patients receiving resin microspheres: D70-Pre-C_MRI_ (before SIRT) = 68 Gy (32–84); D70-Post-C_MRI_ (after SIRT) = 39 Gy (19–80); *p* = 0.02. Bland–Altman analysis confirmed a significant disagreement between these two measurements: with a mean difference of − 16 Gy (95% confidence interval = − 29.6 to − 2.3); *p* = 0.02. There was no other disagreement according to Bland–Altman analysis, between pre- and post-treatment dose metrics.

**Table 3:** Comparison of dose metrics based on imaging before SIRT (^99m^Tc-MAA SPECT/CT; pre-treatment dose metrics) and after SIRT (^90^Y-microspheres PET/CT; post-treatment dose metrics) in patients receiving resin and glass microspheres

	Resin microspheres (*n* = 25)			Glass microspheres (*n* = 23)		
	Pre-treatment	Post-treatment	*p*	Pre-treatment	Post-treatment	*p*
D70-Pre-C_SPECT_ and D70-Post-C_PET (Gy)_	82 [48–106]	52 [34–96]	NS	100 [71–123]	121 [102–166]	NS
Dm-Pre-C_SPECT_ and Dm-Post-C_PET (Gy)_	137 [69–172]	79 [55–150]	NS	150 [107–205]	197 [150–264]	NS
D70-Pre-C_MRI_ and D70-Post-C_MRI (Gy)_	68 [32–84]	39 [19–80]	0.02	91 [40–148]	98 [37–202]	NS
Dm-Pre-C_MRI_ and Dm-Post-C_MRI (Gy)_	115 [108–204]	98 [50–130]	NS	153 [97–241]	156 [94–331]	NS
TNR-Pre-C_MRI_ and TNR-Post-C_MRI_	7.7 [4.6–12.8]	6.9 [3.2–10.2]	NS	5.00 [2.3–8.6]	4.41 [3.1–7.5]	NS

All values are median (*Q*1–Q3). *p* values are based on Wilcoxon test

Good correlation was found between pre-treatment and post-treatment dose metrics, except for scintigraphic-based dose metrics with glass microspheres, and TNR with both microspheres (Table 4).

**Table 4:** Spearman correlation between dose metrics calculated before (based on ^99m^Tc-MAA SPECT/CT; pre-treatment dose metrics)) and after SIRT (based on ^90^Y-microspheres PET/CT; post-treatment dose metrics) in patients receiving resin microspheres (*n* = 25) and glass microspheres (*n* = 23)

	Pre-treatment dose metrics	Post-treatment dose metrics	Resin microspheres (*n* = 25)		Glass microspheres (*n* = 23)	
			*r*	*p*	*r*	*p*
Scintigraphic-based contours	Dm-Pre-C_SPECT_	Dm-Post-C_PET_	0.85	< 0.0001	0.41	NS
	D70-Pre-C_SPECT_	D70-Post-C_PET_	0.81	< 0.0001	0.37	NS
MRI-based contours	Dm-Pre-C_MRI_	Dm-Post-C_MRI_	0.84	< 0.0001	0.82	< 0.0001
	D70-Pre-C_MRI_	D70-Post-C_MRI_	0.82	< 0.0001	0.81	< 0.0001
	TNR-Pre-C_MRI_	TNR-Post-C_MRI_	0.48	0.015	0.35	NS

### Comparison of MRI-based and scintigraphic-based dose metrics

Concerning the comparison of dose metrics calculated with scintigraphic-based contouring and MRI-based contouring (Table 5), there were no significant differences.

**Table 5:** Comparison of dose metrics based on scintigraphic contours and MRI contouring in patients receiving resin and glass microspheres

	Resin microspheres (*n* = 25)			Glass microspheres (*n* = 23)		
	Scintigraphic-based contours	MRI-based contours	*p*	Scintigraphic-based contours	MRI-based contours	*p*
D70-Pre-(C_SPECT_ and C_MRI_) _(Gy)_	82 [48–106]	68 [32–84]	NS	100 [71–123]	91 [40–148]	NS
Dm-Pre-(C_SPECT_ and C_MRI_) _(Gy)_	137 [69–172]	115 [108–204]	NS	150 [107–205]	153 [97–241]	NS
D70-Post-(C_PET_ and C_MRI_) _(Gy)_	52 [34–96]	39 [19–80]	NS	121 [102–166]	98 [37–202]	NS
Dm-Post-(C_PET_ and C_MRI_) _(Gy)_	79 [55–150]	98 [50–130]	NS	197 [150–264]	156 [94–331]	NS

All values are median (*Q*1–*Q*3). *p* values are based on Wilcoxon test

Good correlation was found between scintigraphic-based and MRI-based dose metrics, except for D70-Pre-C_SPECT_ and D70-Pre-C_MRI_ for resin microspheres and D70-Pre-C_SPECT_ and D70-Pre-C_MRI_ for glass microspheres (Table 6).

**Table 6:** Spearman correlation between dose metrics calculated based on scintigraphic or MRI contourings, in patients receiving resin microspheres (*n* = 25) and glass microspheres (*n* = 23)

	Scintigraphic-based contours	MRI-based contours	Resin microspheres (*n* = 25)		Glass microspheres (*n* = 23)	
			*r*	*p*	*r*	*p*
Pre-treatment dose metrics	Dm-Pre-C_SPECT_	Dm-Pre-C_MRI_	0.70	0.0001	0.71	0.0002
	D70-Pre-C_SPECT_	D70-Pre-C_MRI_	0.22	NS	0.47	0.02
Post-treatment dose metrics	Dm-Post-C_PET_	Dm-Post-C_MRI_	0.76	< 0.0001	0.54	0.007
	D70-Post-C_PET_	D70-Post-C_MRI_	0.49	0.01	0.15	NS

### Prediction of tumor control and outcome

Among the 48 patients, median OS was 15 months [IQR 6–22 months], 13 patients had a tumor progression within 6 months and 25 a tumor control (others had a too short follow-up or no follow-up).

Only high (supramedian) TNR estimated before and after SIRT (TNR-Pre-C_MRI_ and TNR-Post-C_MRI_) was predictive of tumor control at 6 months: OR = 5.9 (95% CI 1.3–27.3; *p* = 0.02) and 7.1 (95% CI 1.5–33.0; *p* = 0.01), respectively. Given their redundancy, these two parameters were not associated in a multivariate model.

**Fig. 5:**
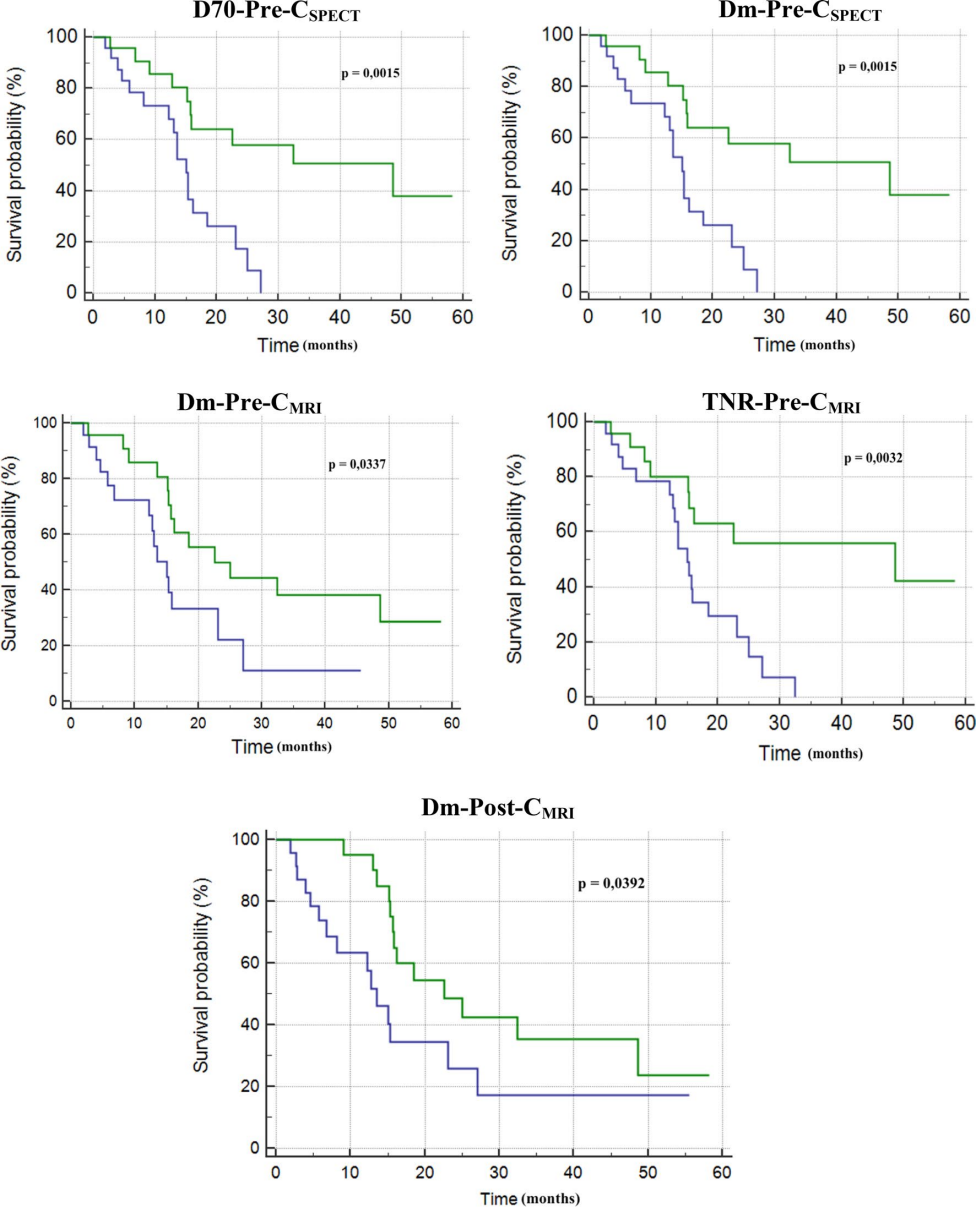
Kaplan–Meier curves on overall survival according to inframedian (blue curve) or supramedian (green curve) Dm, D70 and TNR

Concerning the prediction of survival, high (supramedian) D70-Pre-C_SPECT_, Dm-Pre-C_SPECT_, Dm-Pre-C_MRI_, TNR-Pre-C_MRI_ and Dm-Post-C_MRI_ were able to provide prognostic stratification (Fig. 5). Same results were found according to Cox analysis (Table 7).

**Table 7:** Predictive factors of overall survival. All dose metrics were dichotomized according to supramedian

Pre-treatment dose metrics	Univariate analysis		Multivariate analysis	
	OR [CI 95%]	*p*	OR [CI 95%]	*p*
D70-Pre-C_SPECT_	3.7 [1.6–9.1]	0.003		NS
Dm-Pre-C_SPECT_	3.7 [1.6–9.1]	0.003		NS
D70-Pre-C_MRI_	1.4 [0.7–2.9]	NS		
Dm-Pre-C_MRI_	2.3 [1.04–5.0]	0.04		NS
TNR-Pre-C_MRI_	3.3 [1.4–7.7]	0.005	2.9 [1.2–7.1]	0.02

Post-treatment dose metrics	Univariate analysis			
	OR [CI 95%]	*p*		
D70-Post-C_PET_	1.9 [0.9–4.0]	NS		
Dm-Post-C_PET_	2.0 [0.9–4.2]	NS		
D70-Post-C_MRI_	1.5 [0.7–3.1]	NS		
Dm-Post-C_MRI_	2.2 [1.02–4.5]	0.04		
TNR-Post-C_MRI_	2.0 [0.9–4.3]	NS		

*OR* odds ratio, *CI* confidence interval

Concerning pre-treatment dosimetry, high (supramedian) D70 and Dm obtained with scintigraphic contouring (D70-Pre-C_SPECT_ and Dm-Pre-C_SPECT_) and Dm and TNR obtained with MRI contouring (Dm-Pre-C_MRI_ and TNR-Pre-C_MRI_) were predictive of OS, but only high TNR-Pre-C_MRI_ was independent predictor by multivariate analysis (*p* = 0.02) (Table 7).

Concerning post-treatment dosimetry, only high (supramedian) Dm estimated with radiologic contours (Dm-Post-C_MRI_) (> 98 Gy for resin microspheres and > 156 Gy for glass microspheres) was predictive of survival: median OS = 23 versus 14 months for others (*p* = 0.04) (Fig. 5).

## Discussion

Personalized dosimetry is becoming more and more important in SIRT, especially due to new insights in dose–effect relationships [13, 21, 34] and availability of treatment-planning system for voxel-based dosimetry. However, assessment of accurate absorbed doses remains challenging in SIRT, due to the wealth of technical factors involved in the determination of accurate doses from scintigraphic volumes [35]. Among them, delineation of target volumes appears to be very critical as accurate volumes are a requirement for achieving reliable DVH with voxel-based dosimetry [19]. We evaluated two approaches for target delineation in SIRT. In the first approach, threshold-based contours were automatically delineated on functional images with a fixed 10% threshold chosen to be a reasonable representation of the liver arterial perfusion in SIRT [21, 36]. This approach is supposed to inform us if a simplistic threshold-based delineation leads to similar predictive values to a complete “state-of-the-art” clinical workflow. Indeed, in the second approach, tumors were first delineated on contrast-enhanced MR images and then transferred to the functional imaging modalities (SPECT or PET) after deformable registration.

### Comparison of MRI-based and scintigraphic-based dose metrics

Concerning dose metrics (Table 5), no significant differences were found between threshold-based and MRI-based contours, despite significant volume differences. However, the two contours are natively different: Contours from MRI are representative of the anatomical tumor, whereas scintigraphic contours are representative of the arterial perfusion in the selected area of the liver. Only D70 (and not Dmean) was found to have a weak correlation (Table 6). D70 is expected to be sensitive to heterogeneities and then mismatch between both contours, especially when the tumor exhibits a necrotic part.

Concerning overall survival (Table 7), threshold-based contours from ^99m^Tc-MAA scans were found to be significant predictive factors, whereas those from PET/CT imaging were not. ^90^Y-microspheres PET/CT suffer from count-starving acquisitions and dedicated optimization of reconstruction parameters should be performed to obtain reliable results [19, 37, 38]. Our study uses two different generations of PET/CT systems and lacks of appropriate ^90^Y optimization for the most recent scanner.

MRI-based contours only were predictors of overall survival in both pre- and post-treatment images. Furthermore, TNR derived from these contours appeared to be predictive for tumor control at 6 months as well as for overall survival (in pre-treatment dosimetry). TNR thresholds of 7.7/6.9 (pre-/post-) and 5.0/4.4 (pre-/post-) were identified for resin and glass microspheres, respectively. The range of TNR can be very large for a specific pathology in the literature [39] and will directly depend on the method of delineation. However, with a fixed method of determination, these thresholds could be of real interest to help treatment planning in association with lung shunt fraction and available dosimetric criteria. Although the process used to generate these contours (anatomical delineation plus registration) might appear cumbersome in a clinical workflow, reproducibility was accurate and we believe that this method can be used prospectively to improve accuracy of targets volume determination before dosimetric calculation.

### Comparison of pre- and post-treatment dose metrics

Regarding glass microspheres, no significant differences were found between pre- and post-treatment dose metrics. However, with resin microspheres, D70 was found to be significantly different between pre- and post-treatment images (Table 3). Even with a relative calibration method, PET quantification from small activities usually administered with resin microspheres is challenging and should require a dedicated optimization of the PET reconstruction parameters, as exposed above. Controversial results have been reported in the literature depending on the type of image studied and the method used to assess the correlation between pre- and post-treatment dose metrics [10, 15, 36, 40, 41]. Apart from the technical gesture that has to be strictly identical, technical discrepancies (different kind of particles, injection flow) between the two procedures should explain the large variation of results reported [17] and harmonization of both images (SPECT and PET) should increase their similarity in the context of SIRT [19, 42]. Moreover, as formulated by Lassman et al. [43], the standardization of the SPECT/CT quantification is feasible and should improve results regarding such pre- and post-treatment comparison.

### Tumor dosimetry (MRI-based contours)

Regarding resin microspheres, median values of mean tumor absorbed doses were 115 Gy from ^99m^Tc-MAA scans and 98 Gy from ^90^Y-microspheres PET/CT post-treatment imaging. These results are quite similar with those reported in the literature: In HCC patients treated with resin microspheres, Hermann et al. [13] found a median tumor absorbed dose calculated from ^99m^Tc-MAA scans of 112 Gy with a 100 Gy cutoff identified for longer survival in the SARAH study. Jadoul et al. [16] found tumor absorbed doses to have a median of 69 Gy for ^99m^Tc-MAA scans and 79 Gy for ^90^Y PET/CT. Predictive mean tumor doses of 129 Gy and 135 Gy were also reported by Gnesin et al. [15] and Song et al. [10], respectively.

Regarding glass microspheres, median values of mean tumor absorbed doses were 153 Gy from ^99m^Tc-MAA scans and 156 Gy from ^90^Y PET/CT. Jadoul et al. [16] found tumor absorbed doses to have a median value of 214 Gy for ^99m^Tc-MAA scans and 211 Gy for ^90^Y PET/CT. Regarding dose–response, there is a large consensus in the literature suggesting that a tumor absorbed dose threshold of almost 200 Gy should be appropriate for glass microspheres [21, 44, 45]. Our results, based on a median cutoff, showed that at least 150 Gy was necessary to be predictive of overall survival with MRI-based contours (Table 7). However, large variabilities were also reported in the literature with tumor sizes, heterogeneities and delivered doses being keys influencing factors for the determination of dosimetric thresholds [11].

One advantage of voxel dosimetry is to be able to identify DVH-derived thresholds that could tell us if a certain part of the target volume can be highly irradiated without impairing the organ function. D70 was first proposed by Kao et al. [12] as a DVH-derived dose index capable of accounting for the heterogeneous nature of microsphere distribution. Regarding patients treated for HCC with resin spheres, Kao et al. proposed a threshold of 100 Gy for a complete response. Kafrouni et al. [11] found a mean D70 of 34/45 Gy (pre-/post-) in their cohort with a D70 higher than 80 Gy for complete or partial response at 6 months. Chan et al. concluded that their findings (D70 of 140 Gy for responders vs 24 Gy for non-responders) were comparable to the 100-Gy D70 threshold proposed by Kao et al. though they worked only with glass microspheres. In this study, we found that D70 could be predictive of overall survival but only when threshold-based contours from ^99m^Tc-MAA scans were used. D70 median values of 82 Gy and 100 Gy were identified as predictive for resin and glass microspheres, respectively.

### Limitations

Among the 48 patients included, 23 were treated with glass microspheres and 25 with resin spheres. It has been shown that liver tolerance is quite different between resin and glass microspheres, leading to higher tolerated absorbed doses for glass microspheres in comparison with resin microspheres [46]. To take into account this effect without compromising the statistical power of this study, predictive factors of tumor control and overall survival were assessed by gathering dose metrics from each kind of spheres according to their respective median values.

The retrospective aspect of the study gives us no control to harmonize information and techniques used for each patient individually. However, our cohort was representative of standard recruitment for HCC at our institution and no selection was used to perform data analysis. Concerning post-treatment, dosimetry was carried out on two different generations of PET/CT systems with less than optimal reconstruction parameters for ^90^Y. PET-based voxel dosimetry was performed with a relative calibration method to try to reduce the impact of the use of two different scanners. However, we believe that the use of optimal ^90^Y reconstruction parameters might improve correlations, especially concerning the threshold-based delineation method.

## Conclusion

In this retrospective study, we highlighted the impact of different delineation methods on absorbed dose calculation and predictive factors of tumor control and survival in SIRT. We found good correlations between pre- and post-treatment voxel dosimetry. We confirmed that absorbed dose is predictive of overall survival and showed that TNR is a robust index for prediction, provided that MRI-based delineation is accurate. Due to the different types of images used in a standard clinical workflow, we found that a rigorous process, as made recently approachable with dedicated ^90^Y-microspheres treatment planning systems, could be beneficial for patients in terms of prognostic stratification and should be prospectively considered for management of therapeutic strategy.

## Data Availability

The datasets used and/or analyzed during the current study are available from the corresponding author on reasonable request.

## Abbreviations

^90^Y: Yttrium-90
BCLC: Barcelona clinic liver cancer
BSA: Body surface area
CT: Computed tomography
D70: Minimal absorbed dose delivered to 70% of the tumor
Dm: Mean absorbed dose
HCC: Hepatocellular carcinoma
LDM: Local deposition model
LEHR: Low energy/high resolution
MAA: Macro-aggregated albumin
mRECIST: Modified response evaluation criteria in solid tumors
MRI: Magnetic resonance imaging
OS: Overall survival
PET: Positron emission tomography
PFS: Progression-free survival
PM: Partition model
RMS: Root-mean-square
ROI: Region of interest
SIRT: Selective internal radiation therapy
SPECT: Single photon emission computed tomography
TACE: Transarterial chemoembolization
TNR: Tumor-to-normal liver uptake ratio
TOF: Time of flight
VDK: Voxel dose kernel
